# Endometrial Stem/Progenitor Cells: Prospects and Challenges

**DOI:** 10.3390/jpm12091466

**Published:** 2022-09-07

**Authors:** Caroline E. Gargett, Dharani Hapangama

**Affiliations:** 1 The Ritchie Centre, Hudson Institute of Medical Research, 27-31 Wright Street, Clayton, VIC 3168, Australia; 2 Department of Obstetrics and Gynaecology, Monash University, 246 Clayton Rd., Clayton, VIC 3800, Australia; 3 Department of Women’s and Children’s Health, Institute of Life Course and Medical Sciences, University of Liverpool, Liverpool Women’s Hospital, Crown Street, Liverpool L8 7SS, UK

## 1. Introduction

The human endometrium is one of the most regenerative tissues in the body, undergoing over 400 cycles of menstrual shedding and regeneration during reproductive life. Each month, approximately one centimeter of mucosal tissue—comprising glands and vascularized stroma—is regenerated. This phenomenon is not shared by any typical model organism which undergoes sexual reproduction, and is likely mediated by endometrial stem/progenitor cells. Despite this, knowledge of endometrial stem/progenitor cell biology lags behind other organ systems. During the last two decades, several types of stem/progenitor cells have been identified and characterized: endometrial mesenchymal stem cells (eMSCs), endometrial epithelial progenitor and side population (SP) cells.

This Special Issue, entitled “Endometrial Stem/Progenitor Cells: Prospects and Challenges”, comprises 13 publications from investigators examining the role of endometrial stem/progenitor cells in several gynecological diseases [1,2,3,4,5]. It also provides new insights into aspects pertinent to their identification, function, evaluation and potential for clinical translation [2,6,7,8]. New understanding on the endometrial structure of the unshed basalis layer, comprising a complex of horizontal interconnected glands from which vertical glands regenerate to form the new functionalis layer, suggests a re-evaluation of endometrial stem/progenitor cell niches, and a further understanding of how they function, is now warranted [3,9]. The application of new technologies including endometrial epithelial organoid cultures [3,4,10] and next-generation sequencing at the single-cell level [2] will advance knowledge on the role of endometrial stem/progenitor cells in endometrial biology and proliferative conditions such as endometriosis, adenomyosis and endometrial cancer. Technical advances for improving organoid cultures by generating them from menstrual fluid or from women taking hormonal medications will expand the utilization and clinical translation of this model [10,11]. Studies on the application of endometrial stem/progenitor cells as cell-based therapies and the development of methods for their clinical translation to treat disorders resulting in reproductive failure, including Asherman’s syndrome [3,6,12], recurrent pregnancy failure [3] and other gynecological disorders, such as pelvic organ prolapse [13], are showing promise.

## 2. Endometrial Mesenchymal Stem Cells (eMSCs)

Endometrial MSCs and their perivascular niche identified in the basalis and functionalis (shed during menstruation) layers are the most characterized of human endometrial stem/progenitor cells. Various perivascular locations within the walls of different vessel types have been described based on identified surface markers [6]. The role of perivascular eMSCs in endometrial regeneration is ascribed to their angiogenic, stem cell and immunomodulatory properties. A newer marker of perivascular eMSCs is the exoenzyme NTPDase2, which co-localizes with SUSD2 [1]. NTPDase2 marks multiple cell types in the human endometrium, including basalis stromal cells and ciliated glandular epithelium [1], reducing its value as a marker for purifying eMSCs. The single-cell RNA sequencing of freshly isolated endometrial stromal cells from proliferative-stage endometrial biopsies has identified 10 stromal and 2 pericyte subpopulations [2]. The classic pericyte marker, *MCAM* (CD146), and perivascular markers *SUSD2*, *PDGFRB* (CD140b) and *THY1* (CD90), were generally found in the same individual cells across the two pericytes, and to some extent in the THY1^+^ and ACTA2^+^ clusters. Proteins of these genes have all been used to enrich clonogenic eMSCs with functional stem cell and MSC properties. Transcriptional heterogeneity in these eMSC populations indicates the complexity of the perivascular niche, and may also represent differences in cell state and progression to differentiation. Although this study provides new markers for identifying perivascular cells [2], none are surface markers that could be used to purify the eMSC population. It further confirms that the classic MSC markers, e.g., CD90, do not distinguish fibroblasts from perivascular eMSCs, and neither does their common immunomodulatory gene expression profile. To date, the functional stem cell properties and perivascular niche remain the distinguishing features of these two cell types.

## 3. Histoarchitecture of Human Endometrium

Two reviews on the 3D histoarchitecture of human endometrial glands provide new insights into endometrial physiology, raising important questions on the role of endometrial epithelial progenitor cells in regenerating the functionalis glands each month [3,9]. The discovery of the basalis mycelium-like/rhizomic network of interconnecting horizontal glands, rather than blind-ended tubes, redefines the basalis niche of the endometrial epithelial progenitor cells. It is now important to delineate the in vivo function of previously identified N-cadherin^+^ epithelial progenitors in the branching glands nearest the myometrium and the SSEA-1^+^SOX9^+^ progenitors that are more proximal and near to the functionalis in the endometrial regeneration process. Questions have been raised on how each of these progenitors generate the vertical glands that arise from multiple regions of the same rhizome to generate a region of monoclonal functionalis glands [9]. These mycelium-like branchings provide a structural advantage in preserving epithelial progenitor cell niches during menstrual breakdown, but it is unclear when they arise. During fetal growth or at puberty? They are preserved in the postmenopausal endometrium, but are they altered in endometrial pathologies? Are they present in all regions of the endometrium, as well as in other menstruating species, such as the Old-World primates?

## 4. Endometrial Epithelial Organoids for Investigating Endometrial Stem/Progenitor Cells

Endometrial epithelial organoids (EMOs) are powerful 3D in vitro models that recapitulate estrogen- and progesterone-mediated molecular changes in epithelial cells during the menstrual cycle [4]. EMOs can be exploited to increase our understanding of the molecular signature of endometrial epithelial progenitor cells and their role in endometrial remodeling. Organoids have now been established from decidualized and post-menopausal endometrium, from shedding endometrial tissue in menstrual fluid and biopsies from women taking hormones. Menstrual fluid and hormone-treated epithelial organoids have almost identical transcriptomes to classic EMOs [4,10]. Menstrual fluid offers a non-invasive source of endometrial tissue for generating organoids that could be utilized for personalized medicine. A technical improvement would be to replace the 3D matrix, Matrigel, for generating the EMOs. A first step has been the supplementation of organoid medium with an extracellular matrix component derived from decellularized porcine endometrium [11]. These organoids support epithelial progenitor growth, as most cells are positive for N-cadherin and SSEA-1. Standard endometrial epithelial organoids are heterogeneous in cellular composition, containing differentiated ciliated, secretory and different hormonally responsive epithelial cells, as revealed by scRNAseq (reviewed in [4]). Estrogen treatment increases the epithelial cells and decreases the stem/progenitor cells in these organoids. Further deep single-cell profiling comparing organoids to freshly isolated epithelial cells is required to gain further insight into the relationships between the epithelial progenitor cells and their progenies in this 3D culture model. Defining the exact supplementation requirements of endometrial progenitor and differentiated cell populations will support a more efficient culture of these cells.

## 5. Endometrial Stem/Progenitor Cells in Gynecological Diseases

### 5.1. Endometriosis

Endometrial pathology associated with abnormal endometrial proliferation may involve endometrial stem/progenitor cells. Endometriosis is the growth of endometrial tissue outside the uterus. One review has explored the role of currently identified endometrial stem/progenitor cells and bone-marrow-derived cells in establishing endometriosis lesions, taking into account the theories of endometriosis pathogenesis [5]. It refines the original stem/progenitor cell hypothesis following the recent discovery of somatic mutations in endometrial epithelium and endometriotic lesions, and puts forward a unified dual stem/progenitor cell hypothesis that N-cadherin^+^ and/or SSEA1^+^ epithelial progenitors acquire cancer-associated mutations, and together with non-mutated SUSD2^+^ eMSCs coordinately initiate endometriosis lesions. eMSCs may generate the stromal vascular component. SP cells are also candidates, as they contain both epithelial and stromal cells with stem/progenitor cell properties [5]. However, more research is required to elucidate the precise roles of these stem/progenitor populations in lesion development and to confirm their migration to extrauterine sites. Organoids have been derived from endometriotic lesions, and their co-culture with eMSCs, together with single-cell transcriptional and somatic mutation profiling in future studies, would clarify the role of endometrial stem/progenitor cells in endometriosis [4].

### 5.2. Adenomyosis

Adenomyosis is the ectopic growth of endometrial tissue deep in the myometrium. Its pathogenesis is unclear. Recent tissue clearing of hysterectomy endometrial tissue and 3D imaging has visualized the downward invasion of horizontal basalis glands into the myometrium to form complex branching lesions [9], providing a new understanding of adenomyosis. Cancer-associated somatic mutations have been found in adenomyosis lesions and eutopic endometrium with evidence of clonal expansion, suggesting endometrial epithelial progenitors may initiate these lesions. Further investigations combining 3D imaging and genomic analysis, as well as adenomyosis lesion organoids are required to uncover the pathogenomic mechanisms involved.

### 5.3. Endometrial Cancer

It is well established that individual markers of one adult stem cell population may also mark other cell types without stem cell activity. Functional assays are necessary for determining the utility of any putative marker. This is illustrated for the perivascular eMSC marker, NTPDase2, an ectoATPase associated with tumorigenesis and cancer progression [1]. NTPDase2 is also a marker of normal endometrial basalis stroma. By immunohistochemistry, NTPDase2 was found as a perivascular marker in the stroma of some endometrial cancers. However, NTPDase 2 was also found in the stroma at the leading edge of endometrial cancer invading the myometrium in low-grade tumors. In high-grade tumors, NTPDase2 immunostaining was lost and the stroma showed a myofibroblast phenotype with desmoplastic changes. This study highlights the need to explore the involvement of these and known endometrial stem/progenitor cells in endometrial carcinogenesis.

### 5.4. Recurrent Reproductive Failure

Recurrent reproductive failure encompasses recurrent implantation failure (RIF) and recurrent pregnancy loss (RPL). Both involve the endometrium and its preparation for embryo implantation and pregnancy establishment. RIF results from the repeated failure of embryos to implant during IVF, likely due to diminished endometrial receptivity. RPL results from implanting faulty embryos into an abnormally permissive endometrium. One review has evaluated studies on the role of eMSCs in RIF and RPL [3]. It highlights a problem with the International Society of Cellular Therapies (ISCT) criteria for MSCs: the criteria do not distinguish between functional perivascular MSCs (i.e., clonogenic) and stromal fibroblasts (non-clonogenic) in the endometrium, menstrual fluid, or other organs. Specific eMSC markers were not used to purify clonogenic eMSCs, suggesting that endometrial stromal fibroblasts rather than eMSCs show the abnormal expression of endometrial receptivity markers contributing to RIF [3]. In RPL, SUSD2^+^ (W5C5^+^) eMSCs showed reduced clonogenicity compared with controls. Virtually no data exist on the role of epithelial progenitor cells in RIF or RPL. The luminal epithelium containing a SSEA-1^+^SOX9^+^ epithelial progenitor cell population, which also express the stem cell marker LGR5 may have a critical role in the implantation process, and further investigations are recommended [3].

## 6. Endometrial Stem/Progenitor-Based Therapies

### 6.1. Recurrent Reproductive Failure—Thin Endometrium and Asherman’s Syndrome

MSC-based therapies are being investigated for treating a thin unresponsive endometrium which fails to support embryo implantation, as well as Asherman’s syndrome, where fibrosis replaces the basalis [3,6,12]. Small animal studies show that the local administration of various MSCs, including placental, endometrial MSCs and menstrual fluid stromal cells, promote angiogenesis, reduce inflammation and adhesions, and improve fertility and endometrial thickness through paracrine action [6]. MSCs from the maternal placenta are derived from eMSCs, providing a non-invasive source of regenerative cells [12]. This promising area of research for these intractable clinical problems warrants further investigation, including research into the use of endometrial epithelial progenitor cells.

### 6.2. Other Gynecological Disorders

Endometrial MSCs are being developed as a bioengineered therapy for treating pelvic organ prolapse (POP) [13]. POP is the herniation of the pelvic organs into the vagina as a result of childbirth injury damaging the pelvic floor support structures. eMSCs incorporated into novel non-degradable mesh or degradable nanofibers for supporting the vaginal wall have been assessed in mouse, rat and sheep models. These locally delivered eMSCs act in a paracrine manner to mitigate the foreign body response to implanted biomaterials through immunomodulating the macrophage response, promoting angiogenesis and the deposition of physiological collagen. The local injection of eMSCs into a birth injury rat model of POP prevention showed similar reparative properties. For clinical translation, eMSC culture expansion methods using a small-molecule TGFβ receptor inhibitor, A83-01, has been developed, maintaining MSC properties. The comparison of SUSD2^+^ post-menopausal and menstrual fluid eMSCs, maternal placental MSCs, and bone marrow and CD34^+^ adipose MSCs using this culture expansion protocol increased the proliferation of MSCs from all cell sources, except for bone marrow. It preferentially increased the clonogenic SUSD2^+^ cell component by preventing the apoptosis and senescence of all endometrial-derived MSCs [7]. This study shows the translational value of endometrial-derived MSCs compared with bone marrow and adipose MSCs.

Another approach for the clinical translation of eMSCs is to use 3D spheroid cultures. Menstrual blood stromal cells cultured in a serum-free medium containing a chemically defined lipid concentrate produced uniform spheroids which improved wound healing in a rat model [8].

## 7. Summary

Human endometrium is a highly proliferative tissue which contains endometrial stem/progenitor cells likely responsible for monthly regeneration. They may also play key roles in disorders related to endometrial proliferation and recurrent reproductive failure. New technologies and concepts including single-cell sequencing, somatic mutations, endometrial histoarchitecture and organoid models open different avenues to further understand the roles of endometrial stem/progenitor cells in endometrial dynamics and endometrial pathologies.
